# Future Trend Forecast by Empirical Wavelet Transform and Autoregressive Moving Average

**DOI:** 10.3390/s18082621

**Published:** 2018-08-10

**Authors:** Qiusheng Wang, Haipeng Li, Jinyong Lin, Chunxia Zhang

**Affiliations:** 1 School of Automation Science and Electrical Engineering, Beihang University, Beijing 100191, China; 2 Beijing Aerospace Automatic Control Institute, Beijing 100854, China; ljiny3771@sina.com (J.L.); zhangchunxia126@163.com (C.Z.)

**Keywords:** future trend forecast, empirical wavelet transform, autoregressive moving average model

## Abstract

In engineering and technical fields, a large number of sensors are applied to monitor a complex system. A special class of signals are often captured by those sensors. Although they often have indirect or indistinct relationships among them, they simultaneously reflect the operating states of the whole system. Using these signals, the field engineers can evaluate the operational states, even predict future behaviors of the monitored system. A novel method of future operational trend forecast of a complex system is proposed in this paper. It is based on empirical wavelet transform (EWT) and autoregressive moving average (ARMA) techniques. Firstly, empirical wavelet transform is used to extract the significant mode from each recorded signal, which reflects one aspect of the operating system. Secondly, the system states are represented by the indicator function which are obtained from those normalized and weighted significant modes. Finally, the future trend is forecast by the parametric model of ARMA. The effectiveness and practicality of the proposed method are verified by a set of numerical experiments.

## 1. Introduction

With the rapid development of sensor techniques and signal processing, a variety of sensors are arranged in a complicated system to monitor its operational states. Each sensor can obtain a set of measured values and each reflects one side of the running system, for example, temperature, humidity, pressure, etc. However, those parametric values often have close or relaxed relationships among each other. Moreover, they are affected by noise or interference and it is difficult to judge the operating states directly by those simultaneously measured signals. At the same time, the engineers and researchers are no longer satisfied with real-time monitoring of the running states of a complex system. They want to predict the future operational trend according to the current and previous states. Although each sensor records a real signal independently, for simplicity and practicality, it is better to synthesize a comprehensive one called indicator function taking all measured signals into account. Prior to synthesizing the indicator function, random factors and interference in the measured signals must be eliminated effectively. Based on the synthesized indicator function, the future states of the complex system can be forecast reliably.

To forecast the future operational behaviors or states, the autoregressive moving average (ARMA) model can be directly used [1]. ARMA is a high-precision short-term prediction method for time series analysis and offers a simple description for correlated linear, random processes [2,3]. For a linear time-invariant system, the observed data can be expressed by time-series containing historical observations and measurement noise [4]. When using ARMA to fit or predict a signal, an important issue is to estimate the order number of ARMA [5]. ARMA can be considered as the combination of an autoregressive (AR) and a moving averages (MA) model [3,6]. It is more popular than them because it has their advantages simultaneously, although it is more complex than them [7]. In time series prediction, ARMA provides an more effective linear model with the least parameters [8,9,10]. Hence, it is adopted in this paper for the future trend forecast, using the indicator function.

Prior to synthesizing the indicator function, the random noise in the recorded signals should be eliminated [11,12]. Many signal processing techniques have been used, including fast Fourier transform (FFT)-based methods, wavelet transform (WT), empirical mode decomposition (EMD), empirical wavelet transform (EWT) and some improved methods [13,14]. FFT is the most utilized method in extracting features from analyzing stationary signals [13]. However, it is not suitable for nonlinear and non-stationary signals [14]. To overcome the shortcomings of FFT, wavelet transform has been proposed. However, it requires careful selections of the mother wavelet and decomposition levels, in order to obtain a good time-frequency resolution [15,16]. In addition, synchrosqueezed wavelet transform (SWT) can also be used to denoise the acquired dynamic signals reliably [16]. Recently, a methodology based on the SWT, Hilbert transform and Kalman Filter is proposed for parameter identification [15]. Empirical mode decomposition (EMD) is an adaptive data processing method used to extract the mode information of nonlinear and non-stationary time series [17,18,19,20,21]. The major drawback is the lack of explicit mathematical theory and the existence of mode mixing effect [15,16,19]. In particular, EMD may produce too many modes that are difficult to be interpreted [22].

Empirical wavelet transform (EWT) is proposed recently. Compared with EMD, it has a more consistent decomposition and a rigorous theoretical foundations [22,23,24]. It combines the advantages of Fourier analysis and wavelet theory to extract the different modes in a complicated signal [23,25,26]. Yuan et al. applied EWT in modal identification, by combining with the second-order blind identification (SOBI), to improve the identification performance of modal frequencies [22]. Amezquita-Sanchez et al. proposed a new multiple signal classification-empirical wavelet transform (MUSIC-EWT) methodology [27]. It can obtain the individual mono-components according to the actual frequency information [27,28]. In addition, Amezquita-Sanchez et al. used this method to estimate the natural frequencies (NF) and damping ratios (DR) of large structures [28]. Dong et al. also proposed a modified EWT method based on local window maxima (LWM) [29]. It can obtain the meaningful modes by searching the local maxima of the Fourier spectrum in a proper window and determining the boundaries of spectrum segmentations automatically. In this paper, the modified EWT is used to extract the significant modes in the measured signals from a complex system, in order to synthesize the indicator function for future trend forecasts.

The following sections are organized as follows: the main idea and principle of the proposed method is presented and discussed in detail in Section 2. The numerical experiments are performed and their results are discussed briefly in Section 3. Finally, conclusions are drawn in Section 4.

## 2. The Trend Forecast Method

**Figure 1:**
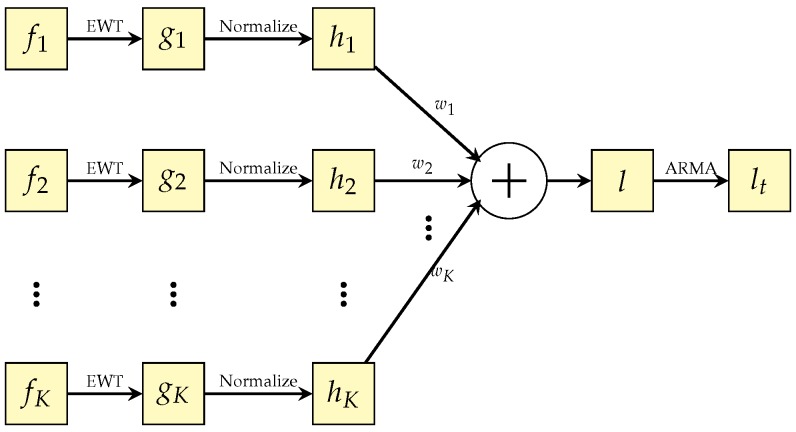
The schematic diagram of the proposed trend forecast method.

In engineering and technical fields, there is a special class of signals that are captured from the same complicated system and have close or relaxed relationships among them. With the system running, each signal only reflects one side of the operational states—for example, temperature, humidity, pressure, etc. If we extract the significant modes of all measured signals, we can synthesize an indicator function, which can be applied to forecast the future operational states of the whole system. Prior to getting the reliable significant mode of each measured signal, the random noise or interference must be carefully reduced, i.e., the extracted significant modes are reliable. In order to achieve the above tasks, we propose a novel method that is based on EWT (empirical wavelet transform) and ARMA (autoregressive and moving average model). The block diagram of the approach is shown in Figure 1.

Suppose $f_{1},f_{2},\cdots ,f_{K}$ are *K* signals captured from a complex engineering system. Each of them is composed of a set of frequency components. The *i*th signal $f_{i}\left(i=1,2,\cdots ,K\right)$ not only includes its significant mode, but also contains other frequency components, naturally including various noise. To extract the significant mode in $f_{i}$, empirical wavelet transform (EWT) is adopted and performed on $f_{i}$, due to its ability of anti-interference and computation effectiveness. Then, the extracted significant mode $g_{i}$ from $f_{i}\left(i=1,2,\cdots ,K\right)$ is normalized into the interval [0,1], to eliminate the impacts resulted from the numerical ranges of the measured signals. The normalized result of $g_{i}$ can be denoted as $h_{i}$, for i=1,2,⋯,K. After that, all significant modes $h_{1},h_{2},\cdots ,h_{K}$ are weighted and summed as the indicator function *l* to reflect the comprehensive operational states of the monitored complex system. Finally, ARMA provides an effective linear model by the least number of coefficients and is performed on the slowly changing *l*, to reliably predict the future operational states of the complex system. The following subsections will discuss the main ideas above reflected in Figure 1 in detail.

### 2.1. Extraction of Significant Modes

In general, the captured signals from a complicated system are inevitably affected by random factors and various noise. If a measured signal has multiple disjoint narrow-band components and wide-band noise, the narrow-band component with maximum relative energy can be considered as the significant mode of the original signal. The significant modes can be effectively extracted from all original signals by empirical wavelet transform (EWT) under the conditions of random interference. EWT essentially designs a set of suitable wavelet filters to get several different bands of a signal (each band corresponds to one mode). In particular, the mode is selected as the significant one due to its outstanding energy. At the same time, the other ones are regarded as the interference components and discarded naturally. In EWT, spectrum segmentation is the most important step to obtain different modes [25,26]. It depends on the reliable detection of the local spectrum peaks of the original signal. In classical EWT, the intermediate frequency value between two consecutive spectrum peaks can be seen as their boundary [20,26]. Suppose the spectrum interval [0,π] of each digital signal is divided into *N* segments and their boundaries are denoted by $\omega_{n}$ ($\omega_{0}=0$ and $\omega_{N}=\pi$). Hence, the empirical wavelet can be constructed by empirical scale function and empirical wavelet function, which are expressed by

(1) $$\begin{array}{c} {\hat{\Phi }}_{n}\left(\omega \right)=\left\{\begin{array}{cc} 1, & \left|\omega \right|\leq \left(1-\gamma \right)\omega_{n},\\ \cos[\frac{\pi }{2}\beta \left(\frac{1}{2\gamma \omega_{n}}\left(\left|\omega \right|-\left(1-\gamma \right)\omega_{n}\right)\right)], & \left(1-\gamma \right)\omega_{n}\leq \left|\omega \right|\leq \left(1+\gamma \right)\omega_{n},\\ 0, & \mathrm{otherwise}, \end{array}\right. \end{array}$$

and

(2) $$\begin{array}{c} {\hat{\Psi }}_{n}\left(\omega \right)=\left\{\begin{array}{cc} 1, & \left(1+\gamma \right)\omega_{n}\leq \left|\omega \right|\leq \left(1-\gamma \right)\omega_{n+1},\\ \cos[\frac{\pi }{2}\beta \left(\frac{1}{2\gamma \omega_{n+1}}\left(\left|\omega \right|-\left(1-\gamma \right)\omega_{n+1}\right)\right)], & \left(1-\gamma \right)\omega_{n+1}\leq \left|\omega \right|\leq \left(1+\gamma \right)\omega_{n+1},\\ \sin[\frac{\pi }{2}\beta \left(\frac{1}{2\gamma \omega_{n}}\left(\left|\omega \right|-\left(1-\gamma \right)\omega_{n}\right)\right)], & \left(1-\gamma \right)\omega_{n}\leq \left|\omega \right|\leq \left(1+\gamma \right)\omega_{n},\\ 0, & \mathrm{otherwise}, \end{array}\right. \end{array}$$

where γ and β(x) are defined by

(3) $$\gamma <\min_{n}\left(\frac{\omega_{n+1}-\omega_{n}}{\omega_{n+1}+\omega_{n}}\right)$$

and

(4) $$\begin{array}{c} \beta \left(x\right)=\left\{\begin{array}{cc} x^{4}\left(35-84x+70x^{2}-20x^{3}\right), & 0<x<1,\\ 0, & x\leq 0,\\ 1, & x\geq 1. \end{array}\right. \end{array}$$

Suppose the *i*th signal $f_{i}\;\left(i=1,2,\cdots ,K\right)$ is processed by EWT, the approximate coefficients can be obtained by the inner product of the signal and empirical scale function:

(5) $$\begin{array}{cc} W_{f_{i}}^{\varepsilon }\left(0,t\right) & =\langle f_{i}\left(t\right),\Phi_{1}\left(t\right)\rangle =\int f_{i}\left(\tau \right)\bar{\Phi_{1}\left(\tau -t\right)}d\tau \\ & =\left(\hat{f_{i}}\left(\omega \right)\bar{{\hat{\Phi }}_{1}\left(\omega \right)}\right)\overset{ˇ}{},\;i=1,2,\cdots ,K, \end{array}$$

where $W_{f_{i}}^{\varepsilon }\left(0,t\right)$ represents the approximate coefficient, $\Phi_{1}\left(t\right)$ represents empirical scale function, and $f_{i}\left(t\right)$ represents the object signal. $\langle f_{i}\left(t\right),\Phi_{1}\left(t\right)\rangle$ represents the inner product of the object signal and the empirical scale function, and $\hat{f_{i}}\left(\omega \right)$ and ${\hat{\Phi }}_{1}\left(\omega \right)$ represent the Fourier transform results, respectively. The symbol $\overset{ˇ}{}$ represents the inverse Fourier transform. Similarly, the detail coefficients of EWT are given by the inner products with the empirical wavelets:

(6) $$\begin{array}{cc} W_{f_{i}}^{\varepsilon }\left(n,t\right) & =\langle f_{i}\left(t\right),\Psi_{n}\left(t\right)\rangle =\int f_{i}\left(\tau \right)\bar{\Psi_{n}\left(\tau -t\right)}d\tau \\ & =\left(\hat{f_{i}}\left(\omega \right)\bar{{\hat{\Psi }}_{n}\left(\omega \right)}\right)\overset{ˇ}{}\;i=1,2,\cdots ,K, \end{array}$$

where $W_{f_{i}}^{\varepsilon }\left(n,t\right)$ represents the *n*-th detail factor, and $\Psi_{n}\left(t\right)$ represents the *n*-th empirical wavelet function. $\langle f_{i}\left(t\right),\Psi_{n}\left(t\right)\rangle$ represents the inner product of the object signal and the empirical wavelet function, $\hat{f_{i}}\left(\omega \right)$ and ${\hat{\Psi }}_{n}\left(\omega \right)$ represent the Fourier transform results, respectively.

The *i*-th signal $f_{i}\left(t\right)$ can be reconstructed by

(7) $$\begin{array}{cc} f_{i}\left(t\right) & =W_{f_{i}}^{\varepsilon }\left(0,t\right)\times \Phi_{1}\left(t\right)+\sum_{n=1}^{N}W_{f_{i}}^{\varepsilon }\left(n,t\right)\times \Psi_{n}\left(t\right)\\ & =\left(\hat{W_{f_{i}}^{\varepsilon }}\left(0,\omega \right){\hat{\Phi }}_{1}\left(\omega \right)+\sum_{n=1}^{N}\hat{W_{f_{i}}^{\varepsilon }}\left(n,\omega \right){\hat{\Psi }}_{n}\left(\omega \right)\right),\overset{ˇ}{} \end{array}$$

where i=1,2,⋯,K. Therefore, the *n*-th empirical modes of each signal can be given by

(8) $${f_{i}}_{0}\left(t\right)=W_{f_{i}}^{\varepsilon }\left(0,t\right)\times \Phi_{1}\left(t\right),$$

(9) $${f_{i}}_{n}\left(t\right)=W_{f_{i}}^{\varepsilon }\left(n,t\right)\times \Psi_{n}\left(t\right),$$

where i=1,2,⋯,K denotes *i*-th measured signal and n=1,2,⋯,N denotes *n*-th empirical mode.

In empirical wavelet transform, FFT is applied to calculate the spectrum of the object signal and then the spectrum peaks are employed to determine the boundaries of the different modes. For a noise-contaminated signal, it may result in incorrect boundaries because the spectrum peaks are sensitive to noise and interference. To improve reliability of spectrum segmentation, the modified EWT, which is based on local window maxima (LWM) [29], is adopted to extract the significant mode of the measured signals in this paper. It can reliably detect the local spectrum peaks at the cost of computation. The main idea of the method is to find all local maximum values of the spectrum and determine the global maximum value as the first peak. Then, all spectrum values around the global maximum value are set to zero. The other spectrum peaks can be found successively in that way until the number of spectrum peaks meets the predetermined requirement. The modified EWT (LWM-EWT) are not sensitive to noise and can void incorrect spectrum segmentation.

**Figure 2:**
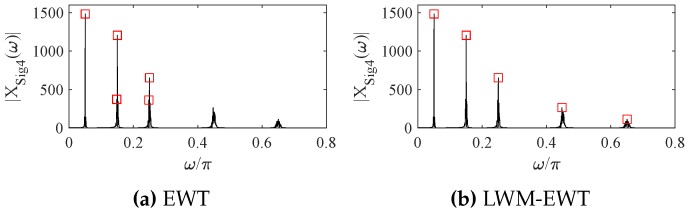
The spectrum peaks founded by the classical EWT and modified EWT (LWM-EWT).

The anti-interference ability of the modified EWT (LWM-EWT) can be verified by the following example. The simulated signal is expressed as:

(10) $$X_{sig}\left(t\right)=\sum_{k=1}^{5}A_{k}\cos\left(2\pi H_{k}\left(f_{0}+\eta \cos\left(2\pi f_{z}t\right)\right)t\right),$$

where $A_{k}$ is the *k*-th value in the amplitude vector $A={\left[1.0,0.9,0.6,0.4,0.2\right]}^{T}$. $H_{k}$ is the *k*-th value of the vector $H={\left[1,3,5,9,13\right]}^{T}$, to tune the *k*-th harmonic frequency. $f_{0}=50$ Hz, η=0.04, $f_{z}=2.1$. The sampling frequency $F_{s}=2000$ Hz and sampling time $T_{s}=1.5$ s. The spectrum peaks obtained by the classical EWT and LWM-EWT [29] are shown in Figure 2.

From Figure 2, LWM-EWT can effectively detect the five significant components contained in the simulated signal and can avoid the excessive spectrum segmentation. Thus, LWM-EWT is adopted to extract the significant modes from the multiple-component signals captured in a complex system in this paper, in order to to obtain more reliable results.

Supposing that *N* modes are obtained by EWT performing onto the *i*-th signal $f_{i}$ (i=1,2,⋯,K), they can be denoted as $f_{i_{n}}$, for n=1,2,⋯N. The relative energy values of *N* modes are calculated by the following equation:

(11) $$P_{n}=\frac{\int_{-\infty }^{\infty }{f_{i}}_{n}^{2}\left(t\right)dt}{\int_{-\infty }^{\infty }{f_{i}}^{2}\left(t\right)dt}\;n=1,2,\cdots ,N.$$

In general, the mode corresponding to the maximum value of relative energy can be considered as the significant one. For a real signal, if five modes are obtained by EWT, their relative energy values are denoted as a vector [0.3,0.5,0.1,0.07,0.03], then the second mode is considered as the significant one, due to its maximum value.

Suppose *K* signals are measured from a complex system, for the *i*-th signal $f_{i}$, the significant mode $g_{i}$ can be extracted by EWT from $f_{i}$, according to the relative energy equation expressed in Equation (11), for i=1,2,⋯,K. Therefore, *K* significant modes can be obtained and denoted as $g_{i}\;\left(i=1,2,\cdots ,K\right)$. The major process of significant mode extraction can be expressed by the following algorithm:

- Load all *K* original signals ($f_{i},\;i=1,2,\cdots ,K$) measured from a complex system.
- For the *i*-th signal $f_{i}$, perform empirical wavelet transform and determine its significant mode $g_{i}$
  *a*. Set the maximum mode number $N_{i}$ and window length $W_{i}$, according to prior knowledge.
  *b*. Calculate the magnitude spectrum of the *i*-th signal by fast Fourier transform (FFT).
  *c*. Use the LWM algorithm to find $N_{i}$ spectrum peaks, using mask window with length $W_{i}$.
  *d*. Calculate $N_{i}-1$ boundary values $\omega_{n}$ for dividing the spectrum, according to $N_{i}$ peaks.
  *e*. Construct $N_{i}$ empirical wavelets with $N_{i}+1$ boundaries, including $\omega_{0}=0$ and $\omega_{N}=\pi$.
  *f*. Perform the constructed wavelet on $f_{i}$ and obtain $N_{i}$ modes, denoted as ${f_{i}}_{n},n=1,2,\cdots ,N_{i}$.
  *g*. For $N_{i}$ modes, calculate the relative energy values, denoted as $p_{n}\;\left(n=1,2,\cdots ,N_{i}\right)$.
  *h*. Select the mode with largest relative energy as the significant one $g_{i}$, corresponding to $f_{i}$.
- Execute the above operations until all *K* significant modes $g_{1},g_{2},\cdots ,g_{K}$ have been output.

### 2.2. Synthesize Indicator Function

For a complicated system, suppose *K* signals $f_{1},f_{2},\cdots ,f_{K}$ from different sensors (temperature, humidity, pressure, etc.) can be recorded simultaneously. Each measured signal only reflects one side of operational states. To reveal overall operating state, all measured signals must be considered. For simplicity, the significant modes are taken instead of the original signals. To minimize the impacts resulted from the numerical ranges, the values of the significant modes $g_{1},g_{2},\cdots ,g_{K}$ must be normalized into [0,1]. The normalization process can be represented by

(12) $$h_{i}=\frac{g_{i}-\min\left(g_{i}\right)}{\max\left(g_{i}\right)-\min\left(g_{i}\right)},$$

where i=1,2,⋯,K. The values of $\max(g_{i})$ and $\min(g_{i})$ denote the maximum and minimum of the significant mode $g_{i}$, respectively. $h_{i}$ denotes the normalized result of $g_{i}$.

Due to the complex correlating or coupling relationships among the measured signals $f_{1},f_{2},\cdots ,f_{K}$, or their significant modes $g_{1},g_{2},\cdots ,g_{K}$, we prefer to look for a comprehensive indicator to reveal the operational state of the complex system rather than consider multiple signals simultaneously. For simplicity, the normalized significant modes $h_{1},h_{2},\cdots ,h_{K}$ are is weighted and summed to make up an indicator function.

(13) $$l=w_{1}h_{1}+w_{2}h_{2}+\cdots +w_{K}h_{K}=\sum_{i=1}^{K}w_{i}h_{i},$$

where $w_{i}$ represents the *i*-th weight coefficient corresponding to the normalized significant modes $h_{i}$, for i=1,2,⋯,K. In particular, they meet the condition of $\sum_{i=1}^{K}w_{i}=1$.

In general, there are two ways to obtain the weight coefficients $w_{1},w_{2},\cdots ,w_{K}$ in Equation (13): one is to derive the weight coefficients according to the accuracy physical model of the complex system. The other is to use the data-driven method to setup the related empirical formula. However, it is very difficult to determine the weight coefficients by these two methods in practice, due to the fact that there are no explicit mathematical models or available empirical formulas to express complicated relationships among the measured signals. For a specific problem, the field engineers may adjust the weight coefficients based on their prior knowledge. Under some unknown conditions, the weight coefficients can be set to the same, i.e. $w_{1}=w_{2}=\cdots =w_{K}=1/K$. It will lead to it being impossible to accurately describe the relationships among the measurement signals, and then lead to inaccuracies of the indication function and predicted results. If the importance of each signals are known, the weight coefficients must be adjusted correspondingly.

### 2.3. Forecast Future Trend

On the basis of the indicator function *l* obtained by (13), we can accurately forecast the trend which reflects the future operational states of the complex system. The autoregressive moving average (ARMA) model is applied to implement this task in this paper. In the theory of ARMA, a measured signal is considered to be a set of random variables that depend on time *t*. Although the individual value which makes up the signal is uncertain, the changes of the entire signal follow a certain rule that can be approximately described by a mathematic model [3,30]. The core idea of future trend forecasts is to use the extrapolation mechanism constructed by ARMA to obtain the better prediction result. The process of ARMA(p,q) can be represented by

(14) $$l_{t}=\sum_{i=1}^{p}\varphi_{i}l_{t-i}+a_{t}+\sum_{j=1}^{q}\theta_{j}a_{t-j},$$

where $l_{t}$ denotes the indicator function *l* depending on time *t*. $\varphi_{i}$ denotes the autoregressive (AR) coefficients at lags *i*. $a_{t}$ and $a_{t-j}$ denote the residual or error terms. $\theta_{j}$ denotes the the moving average (MA) coefficients at lags *j*. *p* and *q* denote the number of AR and MA coefficients, respectively.

From (14), the model of ARMA(p,q) is a memory system that includes the past states and various noise. That is, the sequential value at the certain moment can be represented by a linear combination of *p* historical observations and *q* moving average values of a white noise sequence [4,5]. The AR coefficient $\varphi_{i}\;\left(i=1,2,\cdots ,p\right)$ determines the effect of the historical observations, while the MA coefficient $\theta_{j}\;\left(j=1,2,\cdots ,q\right)$ determines the effect of random factors [3,30]. One step ahead prediction can be represented as

(15) $$\begin{array}{c} l_{t}\left(1\right)=\sum_{i=1}^{p}\varphi_{i}l_{t+1-i}-\sum_{j=1}^{q}\theta_{j}a_{t+1-j}. \end{array}$$

Similarly, L(>1) steps ahead forecast is expressed as

(16) $$\begin{array}{c} l_{t}\left(L\right)=\sum_{i=1}^{p}\varphi_{i}l_{t}\left(L-i\right)-\sum_{j=1}^{q}\theta_{j}a_{t+L-j}. \end{array}$$

Before using the ARMA(p,q) model, we need to test the stationarity of the indicator function, using the Augmented Dickey–Fuller (ADF) criterion. If it is not stationary, the differential transformation is performed until the transformed result is stationary [1]. Then, we determine the orders of the ARMA model for the minimization of the selected criterion function. The criterion functions include the Final Prediction Error (FPE), Akaike Information Criterion (AIC) and Bayesian Information Criterion (BIC), etc.

Another important step is parameter estimation of the ARMA model. In general, the maximum likelihood estimation and the least square estimation can be used to estimate the coefficients of the ARMA model.

In this paper, we determine the parameter ranges of (p,q) using the autocorrelation function and the partial autocorrelation function, which are performed onto the indicator function. Then, we test the ARMA(p,q) model in the valid ranges of (p,q), from low orders to high ones successively. The appropriate model can be determined finally according to AIC criterion proposed by Akaike [31]. In this way, the forecast values can be calculated by (15) and (16), using the indicator function *l* and the selected ARMA(p,q) model. The final results can be considered as the predicted results, which reflect the future operational states of the monitored complex system. In general, the predicted results are evaluated by the percentage of relative errors:

(17) $$\begin{array}{c} V_{error}=\frac{l_{t}-l_{true}}{l_{true}}\times 100\%, \end{array}$$

where $l_{true}$ is the true state value of the complex system.

## 3. Experimental Results and Analysis

### 3.1. Future Trend Forecast of Engine State

**Figure 3:**
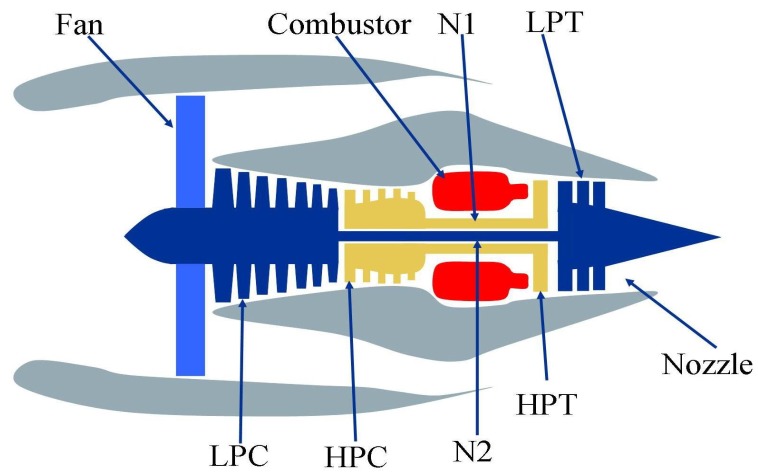
The simplified schematic of the 90 K engine [33].

To verify the effectiveness and practicability of the proposed method in Section 2, we employ the experimental data from the Turbofan Engine Degradation Simulation Data Set that are recorded by several sensors to characterize engine state evolution under different operational conditions [32]. Figure 3 gives the simplified schematic of the 90 K engine. Several sensors are arranged at the High-Pressure Compressor (HPC) to monitor the engine states.

**Figure 4:**
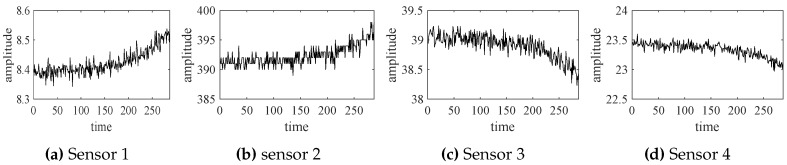
The selected experimental data from four sensors.

The selected experimental data (from four sensor channels) are shown in Figure 4.

**Figure 5:**
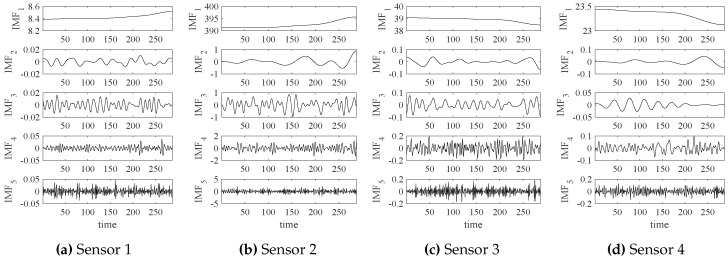
The empirical wavelet transform (EWT) results of four-channel sensor signals.

The empirical wavelet transform (EWT) is performed on those data and the transformed results (IMFs—intrinsic mode functions) are shown in Figure 5.

**Figure 6:**
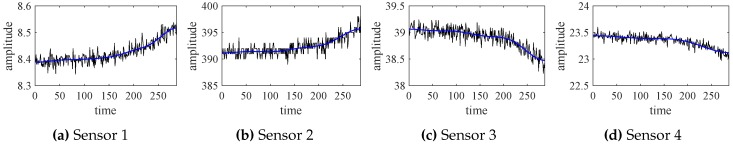
The original signals (black lines) and their significant modes obtained by EWT (blue lines).

Using the formula shown (11), the relative energy values of all IMFs, decomposed from each sensor, are calculated. According to the calculation result, we can find that the first component (IMF${}_{1}$) has much more energy than other IMFs, for all sensor channels. For each sensor channel, IMF${}_{1}$ is selected as the significant mode and the other IMFs are considered as random noise or interference. The significant mode IMF${}_{1}$ and corresponding signal are illustrated in Figure 6.

**Figure 7:**
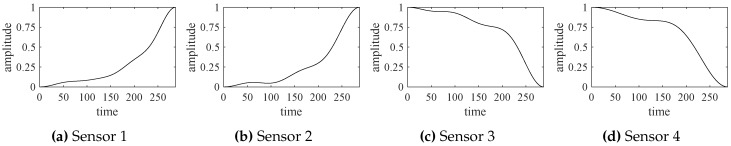
The normalized significant modes of four-channel sensor signals.

According to Figure 6, all significant modes coincide with their original signals very well. That is, they contain the main information from the original signal. Prior to synthesizing the indicator function, the significant modes must be normalized to the interval [0,1], in order to minimize the impacts of the numerical ranges of the sensor signals. The normalized results are shown in Figure 7.

**Figure 8:**
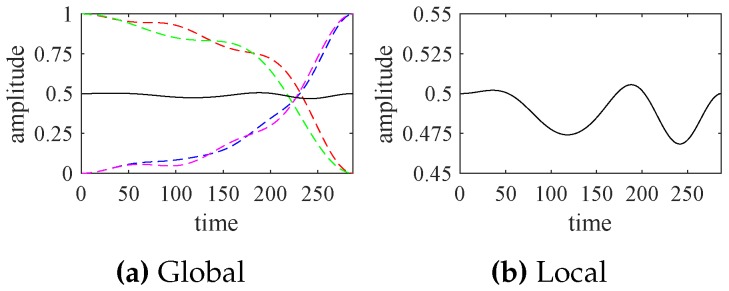
The indicator functions (solid line) and the normalized significant modes (dotted lines).

According to Figure 7, the indicator function, reflecting the comprehensive behavior of the turbofan engine system, is synthesized by (13) and shown in Figure 8. The weight coefficients are set as the same value for all significant modes because we have no explicit mathematical models or available empirical formulas for the engine system. For any complex systems in practice, it is very difficult to accurately determine those weight coefficients, but the field engineers can moderately adjust the weighted values, based on their prior knowledge and experience.

In Figure 8, the indicator function that characterizes the turbofan engine system states slightly fluctuates around 0.5. That is, the engine system is running smoothly or its operating status has no significant changes. In particular, the waveform of the indicator function depends not only on the rise or fall of the significant modes, but also on their increase or decrease slopes.

**Figure 9:**
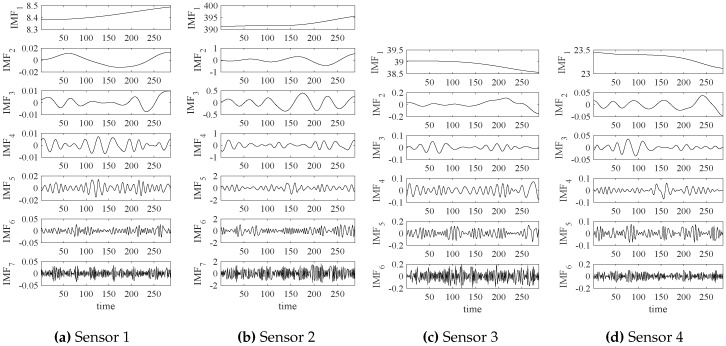
The empirical mode decomposition (EMD) results of four-channel sensor signals.

In addition, empirical mode decomposition (EMD) can also be applied to extract the significant modes of four signals shown in Figure 4. The EMD results of those signals are shown in Figure 9.

**Figure 10:**
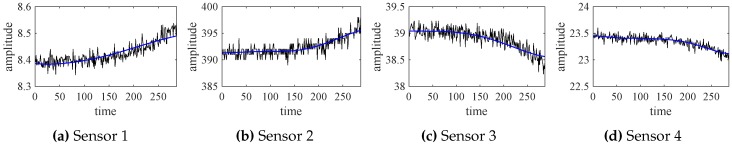
The original signals (black lines) and their significant modes obtained by EMD (blue lines).

Similarly, we can also determine the first components (IMF${}_{1}$) as the significant modes by using (11). The significant modes and original data are illustrated in Figure 10.

**Figure 11:**
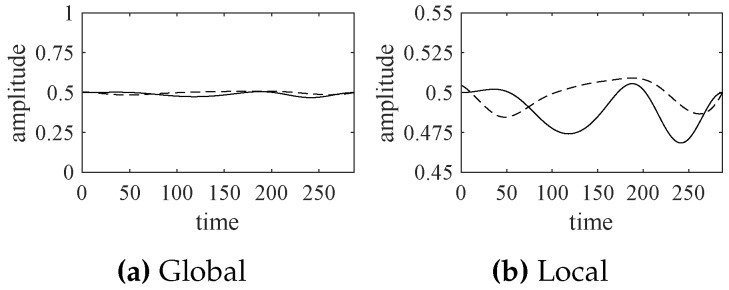
The synthesized indicate functions obtained by EWT (solid line) and EMD (dotted line).

Compared with Figure 6, the significant modes extracted by EWT are a little better than those extracted by EMD. Similarly, we can normalize the significant modes obtained by EMD and synthesize the corresponding indicator function, shown in Figure 11.

According to Figure 11, the indicator functions, obtained by EWT and EMD, are very close to each other. If those indicator functions are applied to forecast the future states of the turbofan engine system, we can obtain similar results.

**Figure 12:**
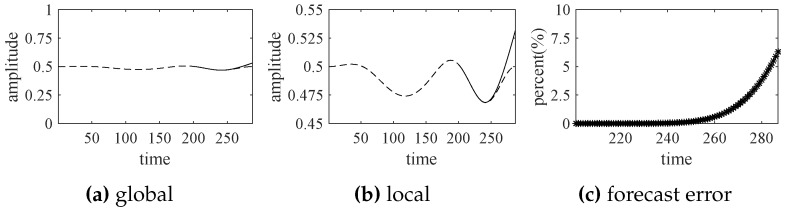
The forecast results by ARMA (solid line) and actual operational states (dotted line).

Based on the indicator function obtained by EWT, shown in Figure 8, the first 200 values are selected as the known data to predict the future values from 201 to 287, using the autoregressive moving average (ARMA) model expressed by (14). The forecast results of the turbofan engine system are shown in Figure 12.

According to Figure 12, the engine system operates normally in the near future (values from 201 to 287) and the forecast trend does not significantly deviate from the actual state curve. The predicted results of this experiment are consistent with the actual situations very well in the short term, although the biases increase over time.

### 3.2. Trend Forecast of Exchange Rates

**Figure 13:**
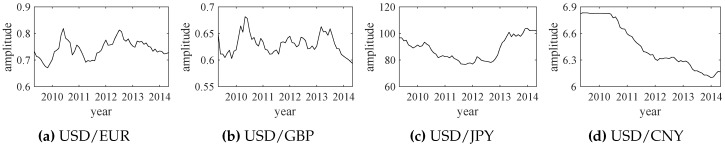
The original data of four exchange rates (from May 2009 to May 2014).

The trend forecast approach proposed in Section 2 can be easily extended to a variety of fields, including science, technology, engineering, society, finance, etc. Here, we use the exchange rate data to verify the effectiveness of the proposed method. The adopted exchange rate data of USD/EUR, USD/GBP, USD/JPY and USD/CN are shown in Figure 13.

**Figure 14:**
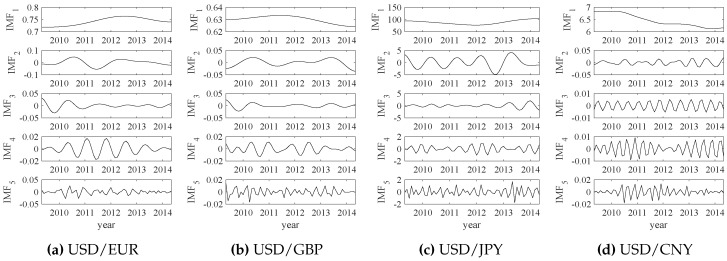
The empirical wavelet transform (EWT) results of the exchange rates.

**Figure 15:**
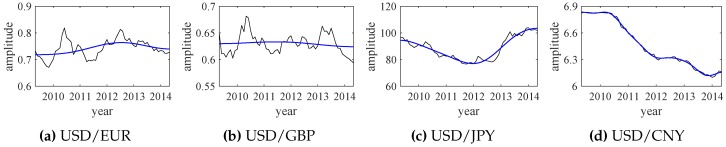
The original exchange rate data (black lines) and their significant modes (blue lines).

**Figure 16:**
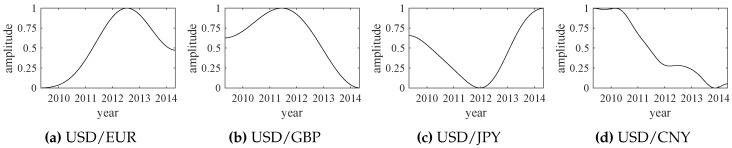
The normalized significant modes of the exchange rate data.

The empirical wavelet transform (EWT) results of those exchange rate data are shown in Figure 14. Similarly, the relative energy values of IMFs are calculated by (11) and all IMF${}_{1}$ components are selected as the significant modes. The extracted significant modes and their corresponding exchange rate data are shown in Figure 15. Each significant mode (IMF${}_{1}$) can represent the overall changes of the original exchange rate data. Then, normalized significant modes are shown in Figure 16.

**Figure 17:**
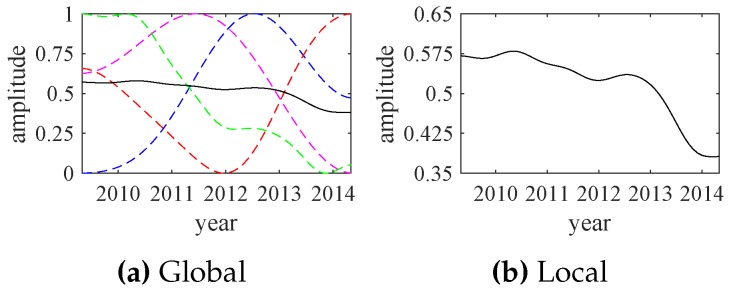
The indicator functions (solid line) and the normalized significant modes (dotted lines).

The normalized significant modes, shown in Figure 16, are applied to synthesize the indicator function of the exchange rates, with the same weight coefficients. The synthesized result is shown in Figure 17. Although the normalized significant modes greatly change over time, the synthesized indicator function changes relatively little. This is consistent with the actual economic and financial developments.

**Figure 18:**
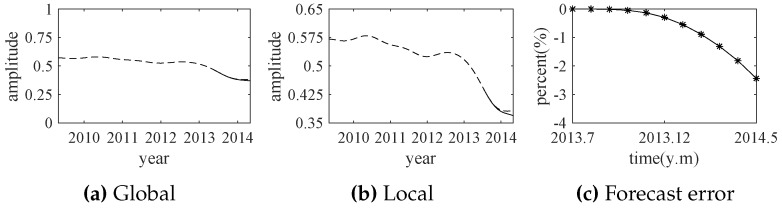
The forecast results by ARMA (solid line) and the actual exchange rates (dotted line).

According to the indicator function, the comprehensive trend of the exchange rates is forecasted by the ARMA model. The forecast results (from July 2013 to May 2014) are shown in Figure 18. The predicted results are well consistent with the actual exchange rates in the short term. This experiment also shows that the presented method is applied successfully in the finance field.

## 4. Conclusions

For any complex system in engineering and technical fields, operational state prediction is a very important technique to guarantee safe operation. On the basis of empirical wavelet transform and the autoregressive moving average model, an effective forecast method is proposed and discussed in this paper. For the multiple signals measured from a complex system, their significant modes are extracted reliably, through taking advantage of empirical wavelet transform and relative energy relationships. Those significant modes are very consistent with the original signal, but they have smoother waveforms or higher signal-to-noise-ratio (SNR). To suppress the negative impacts resulting from numerical ranges, those significant modes are normalized, weighted and summed as an indicator function, which reflects the comprehensive operational state over time. According to the simple indicator function, the future running trend of the complex system is reliably predicted by autoregressive moving average technique. The effectiveness and practicability of the presented method have been verified by a set of experiments whose multiple channel signals were recorded from actual complex systems. The experimental results show that the proposed method has been applied successfully in engineering and financial fields. The proposed approach can also be easily extended to science, technical, social and other fields.

To obtain better forecast results of complex systems, the future work related this paper may focus on: (1) improving the robustness of significant component extraction, and (2) enhancing the accuracy of predict methods.
